# Collision of Fundamental Human Rights and the Right to Health Access During the Novel Coronavirus Pandemic

**DOI:** 10.3389/fpubh.2020.570243

**Published:** 2021-01-08

**Authors:** José Luiz Gondim dos Santos, Paulo André Stein Messetti, Fernando Adami, Italla Maria Pinheiro Bezerra, Paula Christianne G. G. Souto Maia, Elisa Tristan-Cheever, Luiz Carlos de Abreu

**Affiliations:** ^1^Laboratório de Delineamento de Estudos e de Escrita Científica, Centro Universitário Saúde ABC Faculdade de Medicina do ABC (FMABC), Santo André, Brazil; ^2^Cambridge Health Alliance, Cambridge, MA, United States; ^3^Programa de Pós-Graduação em Ciências Médicas, Faculdade de Medicina da Universidade de São Paulo, São Paulo, Brazil; ^4^School of Medicine, University of Limerick, Limerick, Ireland; ^5^Federal University of Espírito Santo, Vitória, Brazil

**Keywords:** coronavirus infections, human rights abuses, right to health, court decisions, jurisprudence

## Abstract

**Introduction:** COVID-19 requires governmental measures to protect healthcare system access for people. In this process, the collision of fundamental rights emerges as a crucial challenge for decision-making.

**Policy Options and Implications:** This policy review analyzes selected articles by the PubMed searcher about extreme measures taken in several countries during precedent pandemics and the current pandemic, and selects hard decisions relating to the exceptional measures taken by judicial departments in Brazil, connecting them to the “collision of fundamental rights and law principles.” The collision of rights and principles imposed on decision makers a duty to provide balanced rights, and to adopt the enforcement of some rights prioritization. Ethical concerns were also verified in this field involving rights limitations. During a pandemic, the importance of extreme measures to protect health rights and healthcare systems is instrumental for focused, fast, and correct decision making to avoid loss of life and the collapse of healthcare systems. The main goals of this research are to discuss the implications and guidelines for public health decision making, the indispensable ethical and legal aspects for safeguarding health systems and the lives of people, and the respect of the Justice principle and of fundamental health and dignity rights. We conclude that COVID-19 justifies the prioritization of collective and individual health access rights. Acceptable standards of fundamental rights restrictions are established at the constitutional and international levels and must be enforced by rules and governmental action, to ensure fast and accurate decision making during a pandemic. Freedom rights exercises must be linked to solidarity for the realization of social welfare, for the health rights of all individuals and for health systems to function well during a pandemic.

**Actionable Recommendations:** All individuals are free and equal, therefore social exclusion is prohibited. Institutions must consider social inequalities when discussing public health measures and be guided by ethical standards, by law principles, and rules recognized by constitutional and international law for the benefit of all during a health pandemic.

**Conclusions:** Collective and individual health rights prevail over the collision of rights when facing pandemic occurrences, case by case, in health systems protection, based on the literature, on precedent pandemics and on legitimate Public Health efforts.

## Introduction

“*Our rights culture cannot constitute us unless all rights count, and all rights cannot count if all rights are absolute.”*^1^

COVID-19 (the new coronavirus disease) requires judicial decision-making and public policies for countries to protect public and private health systems and consequently the well-being, and in a prior way, the health rights of their citizens.

Both at legal and ethical levels, it is desirable that decision-making in public health, which is even more important in a pandemic context, respects the *non-derogable* guidelines of fundamental human rights, also constitutional rights, and respects law-guided ethical standards, in a way to better protect the health rights of people, and to also provide the secure maintenance of healthcare systems.

Extreme measures taken by countries and governments are justified in the context of the novel coronavirus infection pandemic by the fact that the disease has a high transmissibility rate and the methods of transmissibility are not completely understood by scientists.

However, there is scientific evidence that asymptomatic transmissions, for example, are possible at the same rate as symptomatic cases transmissibility. On the other hand, some studies suggest that asymptomatic cases could be less infectious and mainly contribute to the generation of new asymptomatic cases, also having an inferior rate of transmissibility if compared to symptomatic patients (1).

This way, it is not completely defined in what frequency and intensity asymptomatic cases contribute to the pandemic dissemination. In addition, COVID-19 has a critically high rate of contagion that can lead to the potential collapse of healthcare systems, especially as there is no effective treatment or vaccine available for the illness.

It is also quite normal for the hospitalization of critically ill patients to last several weeks (Table 1). A prolonged stay in an ICU facility creates legal, socio-economic, and political consequences that must be considered by governments in regard to healthcare system management in order to keep healthcare systems functioning during the pandemic, and also to avoid, as much as possible, the occurrence of new infections, in the case of shortage of beds in health institutions.

**Table 1:** Median time of admission and hospitalization in ICU of critically ill patients with COVID-19 infections.

**Articles**	**Median time for admission in ICU after symptoms**	**Median time of hospitalization after admission in ICU**
Article (2)	10.5 days	11.5 days or more (survivals)
Article (3)	9 days (survivals) 11 days (non-survivals)	More than 17 days (survivals—at least 3 patients or 5,76% of admitted in ICU)

Source: Table developed by the authors by interpreting articles indicated.

*Articles cited: full description on items (2) and (3) of the references section*.

In this narrated scenario, the collision of fundamental rights emerges as a significant problem for government and healthcare management due to decision making regarding the need to delimit the extension of the acceptable exercise of freedom rights in times of a pandemic. They also need to consider the urgent need for fundamental human rights protection and the implications of pandemic measures on restricting people's rights related to liberty of movement, liberty of travel, liberty of work and to reopen schools.

In addition, more dense social issues demonstrate the illegal conditions of prison facilities and of confinement camps for migrants during the COVID-19 pandemic, that must be considered by authorities to guarantee the right of health for all and to cope with the spread of the virus.

This complex search for better solutions depends on the factual component of the reality of countries and societies. Besides that, communities and states need to attend to important rights for life, health, and human dignity, which are all surrounded by the urgency of public health prioritization.

There are no absolute rights. There are *prima facie* fundamental rights and there are the definitive exercise standards of each fundamental right, in each situation, that are defined beneath the facts and the legal norms of each situation under the law. All of the fundamental rights must be balanced to achieve the maximum protection for all fundamental rights in the reality of social life conflicts and in constitutional principles collision (4).

Post Second World War, European and Western countries, under directives from the United Nations (UN), built legal systems to face the barbarities committed by modern societies. The rights of life and human dignity were elevated to the top of the hierarchy of fundamental rights in constitutions, followed by the rights of freedom, which are also of extreme importance (5).

## Policy Options and Implications

### The Discussion of Balancing Rights to Freedom and the Necessary Decision to Prioritize the Fundamental Human Right to Health in the Context of the COVID-19 Pandemic

#### The Data Selection: Scientific Articles From PubMed Searches and Other Materials From Web-Based Public Data

Articles were selected from the PubMed database due to its large index and collection of world-leading research including scientific journals in health sciences detailing studies on before and after the SARS and MERS outbreaks.

Subsequently, filtering the database was accomplished by searching using the advanced search option in the PubMed website, with no restriction on language, and with the “date-completion” option set between 2003 and May 21, 2020. We used three sets of descriptors, one set for each search, with a total number of three searches, always within the same date range and only substituting the set of descriptors for each search.

The sets of descriptors used were MESH terms: “*coronavirus infections and human rights abuses;” “coronavirus infections and right to health;” and “coronavirus infections and court decisions.”* The three searches returned, respectively, 2, 26, and 167 articles, totaling 195 articles for consideration. We conducted a search using all the descriptors together but this failed as no results were returned.

The method of analysis we applied was to read the titles and abstract of the articles found within the search described and, subsequently, make a choice as to whether the article should be included in the research. We chose articles that were strongly related to the theme of the present manuscript from the authors' point of view and that matched the designed structure of our research.

For that intent, focusing on specific descriptors that could return important articles related to the theme of this research, we established the three sets of descriptors because we found it important within MESH terms to select *coronavirus infections and human rights abuses*, imagining possible violation aspects in restricting rights within the pandemic. Next, we selected *coronavirus infections and rights to health*, health rights are the main rights to be balanced with priority in the face of other fundamental rights in the context of a pandemic.

For the last descriptor, we decided to include *coronavirus infections and court decisions* aiming to find different approaches to guide decision-making during a pandemic considering fundamental human rights and the rights concerning health prioritization.

Other scientific, journalistic, and opinion articles were included in the bibliography and used to develop the discussion within the research, in addition to the selected scientific articles from the searches performed as described above.

The PubMed material was complemented with data scraping carried out by Boolean operators performed in the Google free database before June 30, 2020. We used the Portuguese terms for *coronavirus, COVID-19, court decisions, lockdown, quarantine, social distancing, prison, rules, legal acts, and jurisprudence*. The selection criteria was based on the relevance of findings in regard to the focus of the present article.

These searches found references to Brazilian judicial court decisions in the scope of the COVID-19 pandemic, which were selected, read, and prepared for inclusion in the text. The authors utilized specific searches for judicial court decision numbers on the sites of the courts, as cited in **Table 3**.

#### The Points of View of Scientific Articles on Collision of Rights, Decision-Making, and Priority of the Right to Health During a Pandemic

The articles using the PubMed search engine can be seen in Table 2 to provide a better understanding of the findings. The main findings of the articles found on the theme of pandemic measures are set in this chapter. For example, a research paper on the scope of the Ebola pandemic only recommended quarantine in cases supported by scientific evidence justifying the balance between public security and human rights (6).

**Table 2:** PubMed articles selected and its findings.

**PubMed articles Selected**		**Collision of rights**	**Governments and institutions decisions**	**Defense of health rights prioritization or not**
6	USA	Public security and human rights	Quarantines and isolation	Only in cases supported by science.
				Quarantine if individuals are asymptomatic and the disease is transmissible before the symptoms appear, but only when benefits outweigh risks. Isolation only when individuals exposed are symptomatic.
7	China	Right to movement and public health efforts	Lockdown	Necessity to effective epidemic control at the location.
8	China	Right to movement and public health efforts	Lockdown	Containing the epidemic in the city.
9	China/UK	Public health efforts and human rights	Lockdown, street closures and travel restrictions	Defends that measures are ineffective to health and are against international human rights rules.
11	Canada	Travel economic rights and public health restrictions	Air trafficking restrictions	Defends that measures were unjust, didn't listen to economic sector and didn't distribute Fairly the burdens.
12	Canada	International right to health and public health efforts	Measures to avoid virus spread	China varies from complying (SARS) to not complying (HIV/AIDS) international right to health determinations.
13	Canada	Travel and tourism sector rights and public health surveillance	Travel restrictions	Arguments that surveillance on health care systems entry would be more effective than travel restrictions (suggest pandemic would be less widespread, end sooner and easily contained).
14	Singapore	Public health efforts, social rights and privacy rights	City closure, quarantines, contact tracing and temperature checks	Defends that besides effectiveness of draconian measures to contain the viruses, it is necessary to consider social impacts on privacy and people's rights, also considering legislative history and biomedical science.
15	Singapore	Public health efforts and rights to movement	City closure, quarantines, contact tracing, temperature checks	SARS is controllable and government measures were effective to control virus spread.
16	Canada	Freedom/Objection of Conscience	Health professionals refuse to attend to infectious diseases	There is a threshold that permits health professionals not to take personal risks and always will be volunteers to do the work.
17	United Kingdom	Collective health rights and individual health rights (Pandemics Scope)	Dehospitalization of long term internment with low health injury	Judicial decision defined that the patient should be dehospitalized to make hospital room for COVID-19 injured patients.
18	Tunisia	Freedom rights and government health based rights restrictions	Restrictions measures by government due to the pandemic	Government has the right and duty to take restrictive measures on freedom rights exercise in a pandemic context, based on the human rights order, the Constitution, the civil and administrative law.
19	China	Right to liberty (against arbitrary/illegal penal punishment) and rights to health	Penal law elaboration and punishment of acts during COVID-19 pandemics	Defends penal enforcements prevailing over liberty rights exercise during pandemics to punish health measures Infringements.
22	Switzerland	Liberty to work as a liberal health professional and rights to health	Determination to closure of health professionals clinics, permitted only emergency care	Measure unique for all health professions, difficulty to define emergency cases, the measure doesn't attend the need of health materials economy, and the measure can increase healthcare crisis by letting patients without necessary attendance.
23	Portugal	Right to liberty and public health	Restrictive, exceptional internment and isolation Measures	Restrictions in these cases, in a pandemic scenario, is intended to contribute to the liberty and health of all. Refusing isolation threatens liberty and health of other citizens.
24	Spain	Liberty and freedom rights, public health and right of access to information	Lockdown, quarantines, exceptional restrictive measures	Scientists claimed to be protected the healthcare system with restrictive measures to liberty rights exercise, and with conceding access to COVID-19 data for better formulating scenarios and possibly interventions in the benefit of public health.
25	USA	Right to liberty, right to social care, protection of the right to migrate and right to free health care	Liberty restriction measures	Liberty restriction measures should be mandatory. Besides, governments should do more specially for the vulnerable, guaranteeing healthcare to migrants, also vaccine and effective treatments, once it exists, free of charge to the population.
26	USA/Canada	Liberty to travel and right to health	Restrictions on air trafficking	Authors argue that air travel restrictions are contrary to the norms of international law. (The article 43 of IHR (International Health Regulations) of the WHO (World Health Organization) does not forbid the air travel restriction, “but such measures shall not be more restrictive of international traffic and not more invasive or intrusive to persons than reasonably available alternatives that would achieve the appropriate level of health protection”). Travel restrictions in past outbreaks were of limited Public Health effectiveness. The necessity of travel bans must be weighed against less restrictive alternatives. Social distancing and contact tracing would be more effective in banning the virus spread. Travel restrictions slowed the spread but not halted it. Governments are always seeking to restrict people's rights. Preventions on the diseases depends on international cooperation and rights protection. Instead of restricting rights States should follow WHO recommendations and practice transparent governance, expand testing capacity, and implement social distancing to protect Public Health. Travel bans unnecessarily provokes economic isolation and rights violation. Freedom rights were infringed with travel restrictions. Instead of travel restrictions States should have isolated people. The world is more secure when countries comply with Public Health necessities and Global Health law.
27	UK/Greece	Migrants rights (fundamental human rights) such as liberty and health rights	Policies of migrants confinement	Such policies of migrants confinement in Europe, and confinement camps, are inhumane.These policies deny migrants human rights such as liberty, health, dignity, work, and so on. This situation makes relevant the urgent need for countries to include universal access to health systems as a right of every human being.
28	USA	Individual freedoms and public health	Exceptional measures based on “Emergency Health Powers”	Due to public health protection the possible measures include compulsory treatments, isolation quarantines, limited liability protections, crisis standards of care for hospitals, powers to test, screen and restrict travel, real time requirements of health materials and products, medications, vaccines, person apprehensions if suspected of infection for treatment and tests for up to 72 hours, confinement of infected persons with clear and convincing evidences. Compulsory Health Power should consider evaluating legal and ethical standards, that should include: 1. significant risk of individuals pose an infectious and dangerous disease; 2. interventions must be likely to ameliorate risks; 3. required least-restrictive necessary means to achieve public health objectives; 4. coercion proportionate to the risk; 5. assessments must be based on the best available scientific evidence, but in emerging crises when science is uncertain it is worth base restrictions on the “precautionary principle”. But emergencies in Public Health do not permit coercion that is indiscriminate, overbroad, excessive or without evidentiary support. Home quarantines when correctly taken are much more protective of individual rights liberty and privacy than off-site restrictive measures.

Another research paper on the span of the COVID-19 pandemic attributed the closure of the city of Wuhan as an effective epidemic control measure at that location. The authors of that study also noted that cases of the virus would have increased over time if individuals who were infected had not been contained by public efforts (7). In the same sense, another research paper concluded that the lockdown measures were responsible for containing the epidemic in the city of Huangshi (8).

There was a critical article on street and road-blocking measures, as well as lockdown decrees, indicating that they were totally ineffective in containing COVID-19. Travel restriction measures imposed on Chinese citizens by other countries were contrary to international rules established by the WHO, according to this cited research (9).

However, the WHO (World Health Organization) International Health Regulations (IHR) from 2005 (10), in article 43, does not preclude state parties to establish restrictive measures to prevent people's entry based on health risk demonstrated with scientific foundations, and respecting its internal law and international agreements, being more restrictive than international measures adopted by the WHO. However, this needs to be communicated to the WHO and a procedure of verification of its maintenance must be adopted after 3 months of the implemented measure.

On the other hand, the previous research indicates that it is common knowledge that it is necessary to contain person-to-person contagion in the scope of the COVID-19 pandemic in a way to reduce the infected number of people, and that this is possible if people maintain social distancing. Additionally, it is especially important to achieve the development of therapies and vaccines based on science to face the pandemic (9).

From an economic perspective, one article finds that it is questionable that there was justice in the restrictive air traffic measures related to Toronto during the SARS epidemic in 2003, which would have imposed a local loss for the Toronto travel industry of approximately 1.1 billion dollars (11). This research considered that the restrictions would not have been ethical because the WHO did not listen to local authorities before the restrictions were imposed and the burden would not have been distributed equally (11).

On the theme of human rights, an essay indicated that China varied between complying with international determinations of the right to health (in the SARS case) and total non-compliance (in the HIV/AIDS case). In the case of SARS, China complied with measures to prevent the spread of the virus; in contrast it did not guarantee that Chinese patients suffering from HIV/AIDS had a right to access possible treatments (12).

According to the authors from the above article (12), rights in China do not have the same nature as the human rights in the international arena and in most Western democratic countries, where rights are considered inherent to every person. In China, there are only concessions of rights made by the government depending on a person's commitment to duties imposed by the government.

The prospects of rights in China, in comparison to other countries ruled by international human rights, are fundamentally diverse, because the rule of international human rights imposes the recognition of rights to every person, without conditions, such as liberty, political rights, dignity, life, health rights, social rights, and cultural and economic rights. Its intensity, extension, and depth vary according to the development stage and richness of a country.

Some dared to predict that if a new epidemic such as SARS appeared, and the local government instead of implementing airport passenger surveillance, affecting the economic travel and tourism sector, had only invested in surveillance at the entrance of health systems, the epidemic would end sooner. The pandemic would be less widespread and more easily contained (13).

Health surveillance and precise restriction measures to avoid public movement in Singapore during the last pandemic demonstrated that fewer people traveling contributed to lower rates of infection. This happened during the SARS epidemic and now has happened during the COVID-19 epidemic (14).

Relating science data to social issues, the research paper demonstrated that measures to restrict and contain an epidemic must follow biomedical criteria and must also consider the social implications of restrictions on rights to movement that affect many people's privacy, liberty, and social rights. The island of Singapore is described to be generally closed during pandemic occurrences with a ring of protection, in conjunction with quarantine, contact tracing, and temperature checking measures in public places (14).

Besides, it was verified through a mathematical model that the interventions of Singapore's government in containing the SARS virus were able to stifle the outbreak of the disease (15). This study did not focus on people's rights collision during the SARS pandemic but tried to answer two questions: was SARS controllable with restriction measures taken by the government and would these measures be effective?

This cited research (15) was successful in proving a positive answer to both questions above. This corroborates the defense of prioritization of collective health rights guaranteed by the measures taken in the SARS pandemic in Singapore. However, the limitation of the study, due to a focus on a mathematical model to prove efficiency of the restriction measures, did not demonstrate a broad view of people's rights in Singapore, which would have been desirable.

Among the possible measures of rights restrictions enacted by governments during a pandemic, there may be attempts to compel health professionals to assist patients with COVID-19 against of their own will (of health professionals). Would that be feasible? This question was raised during the SARS epidemic, where fatality rates were significantly higher than for COVID-19 rates.

The fatality rates of health professionals during both the SARS and COVID-19 outbreak were very high and ethically some health professionals refused to treat infected patients. Could this choice be considered reasonable? In this specific case, because of the rule that permits health professionals to safeguard their own health, the option of not treating infectious patients is justified at least in the context of SARS since there will always be volunteers to do the work, this was the conclusion of other research paper (16).

Judicial decisions on healthcare have social repercussions in the United Kingdom due to the common law system, and the system of precedents (17). An important case mentioned is the case of MB (patient name as initials, not publicized in full in the article for confidentiality reasons) admitted a few years ago to a London hospital.

The hospital sought an injunction to remove the patient from its facility in order to make room for COVID-19 patients. The hospital had already tried to move the patient to a communal home that would meet his needs; however, the patient refused. The judge decided on his removal and prevented him from returning to the hospital without express permission, except if brought by an ambulance [case: University college London Hospitals NHS Foundation Trust v MB [2020] EWHC 882] (17).

In the context of Tunisia (18), the government's right to restrict freedom rights during the exceptional context of a pandemic stems from the international human rights order, the Constitution, and the administrative and civil law normative acts. The same is true in Brazil, and for most state parties in the WHO, and in the same exact order of importance (10).

One research article set in China suggests that local governments must urgently make adaptations to their legal systems with a focus on the penal system, like China has been doing, in order to be able to punish people who fail to comply with restrictive measures aimed at containing the advance of the COVID-19 pandemic (19).

The penalties for these crimes vary from several months of detention to more than 10 years of reclusion. In the article cited (19) the problem discussed is that after the beginning of the COVID-19 pandemic, authorities in China supposedly made some alterations in the penal law so that they could punish people for infringement of measures during the pandemic.

In the Brazilian case, the penal code has a prevision for the so called danger crimes that can be applied to acts that do not adhere with governmental and health measures taken to avoid infections during pandemics, such as articles 131 and 132. The Brazilian penal code also prescribes the epidemic crime (article 267), the crime of infringement of sanitary measures (article 268), and the crime of disobedience (article 330) (20).

Caution is recommended because the main principle of the penal law is the legality principle, according to which the crime and the penalty for its commitment must be prescribed in law before the conduct that will be punished occurs (from the Latin sentence “*nullum crimen nulla poena sine praevia lege”)* (20, 21).

Two additional issues: the criminal approach is different in the Chinese context, for reasons already discussed in this study, and human rights seem not to be fully respected in China compared to the international sphere. Therefore, considering that in criminal facts coercion measures applied to previous facts under the principle of criminal legality cited above, it is not acceptable that the advance of the pandemic can be contained, an intention necessarily directed toward the future, by law enforcement to punish the authors of crimes.

The penal approach to stop the virus spread is only acceptable if it respects the penal principles, the due process of law, fundamental human rights guarantees, and if it is extremely necessary, as the last choice of the authorities to contain individuals conducting imminent and dangerously deliberate spread of the virus. It is the *ultima ratio* legitimate intervention.

In Revue Médicale Suisse (RMS) (22), medical doctors argue that it is not possible to follow the determination of public authorities for non-urgent cases, the so called *fermeture obligatoire des cabinet medicaux per le Ordonnance 2 COVID 19*, with only permission for emergency medical care being maintained.

State restrictions have even created a definition for an urgent situation, such as one that cannot be postponed to another date. However, the authors claim that there are situations in which it is not possible to clearly determine if it is urgent or not, and that even patients whose conditions are originally not urgent can quickly evolve into an emergency that requires immediate intervention (22).

In Portugal there is context for the application of restrictive, exceptional, internment, and isolation measures indicated for reasons of public health, as detailed by constitutional interpretation and jurisprudence. There are no explicit provisions allowing restriction of liberty in the CRP (Constitution of the Portuguese Republic) in the case of a pandemic, but, as these authors explain, in these exceptional circumstances, restrictions are intended to contribute to the liberty and health of all, since the patient that refuses isolation threatens the liberty and health of the other citizens (23).

In the Spanish context of the pandemic, experts asked the authorities to decree a lockdown and to grant access to pandemic data to researchers, because it is indispensable to guarantee the right to information. This can also contribute to the formulation of exceptional measures to face the pandemic based on facts and scientific evidence (24).

Scientists also claimed, in addition, for the authorities to take more restrictive measures on freedom rights for the purpose of containing the advancement of COVID-19 (24). According to these authors, the Spanish government's timid measures were not enough to contain the pandemic's progress, and it eroded the foundations of the Spanish health system (24).

A finding in an article included in this study stated that people should follow the mandatory recommendations and restrictions, and comply with orders of social distancing. Moreover, governments should do their part, especially for the most vulnerable. For example, guaranteeing social and health care to immigrants, who fear deportation and would hide even if they were sick, causing individual and social damage. Governments must guarantee treatment, and, once a vaccine and effective treatments exist, they must be assured free of charge to the population, to avoid inequalities (25).

As a counterpoint to the idea that restrictions on the right to freedom should prevail to contain the advance of the pandemic, and consequently to safeguard the right to health, some authors advocate that restrictions on air travel are contrary to the norms of international law (26). But as we saw above in article 43 of the IHR, measures that restrict people's entry into state parties of the WHO to face health risk are not forbidden but have to be founded on scientific evidence and have to adhere to a procedure of communication and verification.

According to another study (27), policies of confinement of migrants in Europe, mainly in countries with low to medium income, threaten the efforts to contain the progress of COVID-19 in major European centers. Thousands of migrants do not have access to water, soap, medicines, toilets, and electricity, and they are confined to detainment facilities, such as confinement camps, without basic health conditions (27). These inhumane conditions are perfect for COVID-19 transmission, which can increase the rates of contagion in European centers going forward.

Several authorities in countries like Greece have already been informed of the need to eliminate such confinement camps in the scope of the pandemic, but no such measures were taken. Measures to cope with COVID-19, like those policies of the 2030 Agenda, should include universal access to health care systems for all people as an emergent need (27).

Restrictive and social distancing measures do not work and are not possible for confined migrants, in this inhumane scenario. All the efforts to contain COVID-19 could be in vain if there remain migrant confinement camps in the Mediterranean.

In the light of the declaration on January 31 2020 that the COVID-19 pandemic in the United States of America was an emergency and a clear exceptional situation of crisis, the government had a special responsibility to carefully balance the protection of public health and individual freedom (28).

The present article is about unveiling pandemic extreme measures that can be taken by governments and health authorities and that make it possible to better protect rights to freedom and health, guaranteeing human rights protection and promotion in a solidarity and collective way during a pandemic.

### Collision of Rights to Freedom in the Face of the Rights to Life and Health During the Novel Coronavirus Pandemic

#### Brazilian State Policies to Face the Pandemic and the Judicialization of Collisions of Fundamental Rights in Brazil in the Scope of the COVID-19 Pandemic

Fundamental rights in Brazil are based on its Constitution. There is a claim that fundamental freedoms and their exercise by individuals and groups of people collide with the right to health in the scope of the current pandemic of COVID-19, which is similar to what happens in different parts of the world, as described in Table 2.

In the COVID-19 context we must admit, by the data collected (Table 3), that the head of the Federal State of Brazil has adopted contradictory measures during the pandemic. It is also possible to detect that he does not deliberately implement global health recommendations, and rejects any coordination with local governments to cope with the pandemic effects and containment. This can also be demonstrated in the science denial discourse of the head of the State (29).

**Table 3:** Examples of Lawsuits in Brazil in the collision of fundamental human rights during the Coronavirus pandemic.

**Lawsuit proposer**	**Action requests**	**Court**	**Decision**
1. Democratic Work Party (by Brazilian Federal Deputy).	Unconstitutionality of a federal provisional law that removed the competence to impose restrictive measures from Brazilian States and Municipalities.	Brazilian Supreme Federal Court.	Injunction granted to maintain the competence of the Union, States and Municipalities to impose restrictive health measures during the pandemic^1^.
2. Federal State of São Paulo.	To unblock the roads on the coast of Caraguatatuba.	São Paulo State Court of Justice.	Suspension of the injunction and the judgment that blocked the roads^2^.
3. Brazilian Federal District.	Mandatory submission to diagnostic examination and home isolation in one necessary case.	Federal district court of justice.	Injunction granted^3^.
4. Municipality of Ariquemes Rondônia	Injunction and judgment suspension to permit open trade within the city.	Superior Court of Justice.	Suspension not granted^4^.
5. Citizen 1^a^	Preventive *Habeas Corpus* not to be submitted to social isolation.	Superior court of justice.	Injunction not granted^5^.
6. Citizen 2^b^	Home Prison.	Superior court of justice.	Measure approved^6^.
7. Citizen 3^c^	Home Prison	São Paulo state court of justice.	Measure not approved^7^.

Source: Data collected and prepared by the authors in the named courts' databases, after search and scraping data in the Google database with options described in the ‘Data Selection' section above.

*Chart legend: A: Citizen 1 did not want to be hypothetically submitted by a government rule to social isolation in case of infection by the novel coronavirus. The court decision did not permit the citizen avoidance of the health measures adopted by the government. B: Citizen 2 was a male jail prisoner accused of drug trafficking caught with 101 g of crack and 99 g of cocaine and would have committed the crime without violence and was preventively arrested without conviction—overdue preventive detention. The court understood that in exception, because of the COVID-19 pandemic, and the fact that the crime was committed without violence or serious threat, to substitute the institutionalized prison to the home prison measure, according to pandemics guidelines recommended by the CNJ (National Counsel of Justice), prevailing a preventive measure to the health benefit (cope the coronavirus infections) over the criminal procedural law enforcement of the maintenance of the jail prison decree due to the gravity of the crime (great amount of drug apprehended) and general reasoning of jail preventive imprisonment in the protection of the public order. C: Citizen 3 was a female jail prisoner, mother of a child, condemned for drug trafficking with a community service punishment not complied to. This was replaced by arrest for 2 years, and the measure required was not approved due to the judgment consideration of the child's best interest in maintaining distance from the mother. 1: Direct Action of Inconstitutionality (ADI) n° 6341 from the Supreme Federal Court (STF) stf.jus.br, search of law suit tool by class (ADI) and number (6341). 2: Suspension of Injunction and Sentence (SLS) n.° 2054679-18.2020.8.26.0000 from the Court of Justice of the São Paulo State (TJSP) tj.sp.jus.br, search of law suit tool with the number. 3: Civil litigation n.° 0701858 04.2020.8.07.0018 from the Court of Justice of the Federal District (TJDF) tjdft.jus.br, search of consultations tool, public consultations, 1st instance, with the number. 4: Suspension of Injunction and Judgement (SLS) 2697 RO from the Superior Court of Justice (STJ) stj.jus.br, search of law suit tool with SLS 2697 descriptor. 5: Habeas Corpus (HC) 576058 DF 2020/0095453-4 from the Superior Court of Justice (STJ) stj.jus.br, search of law suit tool with HC 576058 descriptor. 6: Habeas Corpus (HC) 564736 SP 2020/0054426-4 from the Superior Court of Justice (STJ) stj.jus.br, search of law suit tool with the descriptor HC 564736. 7: Habeas Corpus (HC) 20602463020208260000 from the São Paulo State's Court of Justice (TJSP) tj.sp.jus.br, search of law suit tool with the number*.

On the other hand, the Senate of Brazil decreed, after the President of the Republic requested, based on the Constitution, a state of public calamity to cope with the pandemic using economic measures. A state of emergency in public health was determined by the Ministry of Health to cope with the pandemic and adopt necessary health measures.

It also established, by law, the possibility of the government adopting, among other measures, the restriction to movement of people, compulsory submission to diagnostic tests, social isolations, quarantines, lockdowns, and the request of private assets for the use of the State. It was also declared by law that all people in the scope of the pandemic have the right to be treated free of charge. These are the main potential measures to be taken by the country's government to face the pandemic (30), at federal and local levels.

State and Municipal governments in Brazil, within the scope of their constitutionally guaranteed competence in health issues, have addressed normative and administrative acts (pandemic measures) to restrict the movement of people, established compulsory isolation of individuals, and have made determinations to carry out diagnostic tests on specific individuals. They likewise established quarantines, social distancing rules, and other restrictions such as road and street blocking, as well as specific lockdown measures.

There have been judicial decisions on conflicts related to the pandemic, and recurrent judicial rulings recognizing the prioritization of the right to health that justifies restrictions in freedom rights. The most important and iconic of the conflicts that have occurred so far was at the legislative initiative of the President of the Republic in which he aimed to prevent State and Municipal governments from adopting measures to restrict the exercise of freedom rights, such as social isolation, quarantine, and local lockdowns.

This was provisionally prevented by the Federal Supreme Court. The Brazilian President tried to avoid local governments having to balance the fundamental rights to health access under their constitutional competency and the freedom fundamental rights, in order to be capable of determining pandemic measures to cope with the spread of the virus, and the Brazilian Supreme Federal Court did not permit that limitation by the President, which would have been terribly unconstitutional.

There was a declaration by the court that State and Municipal governments, as well the Federal government, can legislate on health under the Constitution rules and principles, but especially in the pandemic scope, in order to face the emergency of public health.

Conflicts and collisions of rights have been judicialized in several Brazilian courts. The matters are diverse, such as lawsuits that have been filed to release prisoners from risk groups to avoid the damaging consequences of COVID-19. In this kind of situation, the right to health only sometimes prevailed over the State's right to punish in the precarious context of the pandemic, as demonstrated in Table 3.

There are also cases of compulsory testing and social isolation. In such cases, the right to health has prevailed to the detriment of individual liberties in the cases verified (Table 3)

It is interesting to note that, in a way to prioritize health rights in habeas corpus petitions, in order to maintain social distancing, the claim verified has been that house arrest (relative liberty compared to prison) is necessary for inmates, in the risk group or with special conditions, to serve out their sentences or provisional prison measures.

In a case in which the judicial decision was to concede the mandamus, the court understood that imprisonment during this pandemic conflicts with health rights protection, and so it made the prioritization of health rights (Citizen 2, Table 3).

Some of the most relevant types of lawsuits involving the collision of principles which establish fundamental rights that have been found in the Brazilian pandemic situation are listed in Table 3. The right to health in the majority of the cases selected (5–2) prevailed over the other interests in the context of the pandemic.

#### The Weighting and Balancing of Rights in Collision

There are theoretical guidelines in legal doctrine for making judicial decisions in the face of a collision of law principles and for conflicts of legal rules. On the other hand, in the theories of the field of justice there is a consensus that “A Theory of Justice” from John Rawls is a watershed moment (31, 32).

John Rawls is the theorist that, from the second half of the 20th century onwards, changed the focus of the justice issue in a liberal way, focusing on the fairness of the justice, placing it in a set of rules for the better stand of liberty to all (egalitarian liberty) and of democratic equality completed by the sense of the principle of difference (31, 32).

It is recognized in doctrine that justice was, in Rawls's theory, replaced in a different focus considering the distributive sense of just measures to all, and that Rawls conceived his theory based on criticizing the utilitarianism ethics.

In summary, the justice theories have developed since Plato's concept of justice as happiness of the city and of its guardians, and Aristotle's concept of justice as equity. Subsequently, justice was connected to Hobbes's and Locke's concepts of State and Justice, founded on the power of the strongest due to a necessary obedience of the sovereign, and based on the right to property, respectively.

Justice conception was completed in this chronology by the utilitarian theories of Jeremy Bentham and John Stuart Mill based on the principle of happiness. After this evolution until the 19th century there was not another widely relevant new theory of justice before John Rawls.

At the other side of the current approach of this research, hard decisions in which fundamental law principles that contain the fundamental rights of people collide, Ronald Dworkin sets the problem assuring that rights only apparently collide because constitutional rights, at his notion, are neat and clear concepts that need to be known by the interpreter, from a point of view of the internal theory, without external influence of other fundamental rights, according to the concept that the principles are found in their internal content (4, 33).

But Dworkin accepts the balancing and weighting of the interests involved in such cases, but in a hidden way, with justification deficiency to the theory (33). At his side, Robert Alexy delivered an interpretation of law rules and principles, and in his conception of these optimization commandments (constitutional principles), from an external theory, it is necessary to balance and weigh principles and assume rights collisions, in a construction of a well-accepted technique to solve collision of rights and principles in court and state decisions (4, 33, 34).

Supported by the theory of principles by Robert Alexy, and the concepts of rules and principles by Ronald Dworkin, and also the horizon of liberty and equality borrowed from John Rawls's “A Theory of Justice,” it is possible to discuss the established rules created to face the COVID-19 pandemic in the context of the scenarios given by the selected PubMed searcher articles, in several countries in the world and different legal systems, considering also the Brazilian constitutional context in comparison.

Fundamental rights are essentially relative in the sense that there is no fundamental right, based on a principle, of absolute nature, according to the prevalent interpretation of the Constitution. For Pildes (35), although constitutional theory and political philosophy understand that rights are individual trumps for autonomy, dignity, and liberty against decisions in the common good, constitutional practice indicates that rights function in another sphere rather than acting in atomistic protection of individual interests. For this theorist, rights serve as tools for courts to evaluate the social meanings and dimensions of governmental action. In this way, rights are means of realizing the common good.

The exceptional situation of the COVID-19 pandemic and the need to face the public health emergency make it possible for authorities to balance constitutional principles, and to create and enforce legal rules that impose direct restrictions on the exercise of individual and social rights in the prioritization of the common good. Thus, greater constitutional and democratic values can prevail amid the pandemic.

The rights to life, public health, and human dignity are examples of fundamental rights of unquestionable social and legal importance. Due to the pandemic these fundamental human rights take precedence in a weighting of values in comparison to the mere right to freedom dissociated from the values of solidarity, self-protection, precaution, and care.

For most general liberty exercise rights to prevail over the right to health during a pandemic, they must be linked to solidarity, self-protection, precaution, and care. Examples are the cases of health professionals that refuse to assist COVID-19 patients, or of prisoners in Brazil, or of migrants in Europe or near the Mexican border in North America, whose restrictions to liberty in prison institutions render them unable to protect themselves from the virus spread in the pandemic, which is unacceptable.

These findings are based on the legal system, on constitutionalism, and the normative nature of constitutional principles, and are observed in the literature. In cases of collision of rights, the fundamental rights that carry a social relevant value, notably the rights to health, to liberty with solidarity and self-protection, to equality, protection of all human lives, promotion of human dignity, and social justice efficacy, must be, in all of these cases, prioritized as a necessary respect of the rule of ethics and rule of law that must be necessarily attended.

## Actionable Recommendations

In his Theory of Justice, Rawls (32) locates political action at the encounter between the rationality of the modern political social contract and geometric morality.

In his work “A Theory of Justice,” Rawls (32) assumes that in each society all individuals must be equally free, autonomous, and democratically equal (principles of egalitarian liberty and democratic equality). No one may be subject to discrimination or exclusion, and institutional objectives must move in the direction and primary purpose of poverty reduction and, therefore, of social ills.

This theory of justice is complemented by the principle of difference, according to which institutions must be structured based on the observation of social inequalities, and institutional practice must produce, in the long run and in the future, greater benefits to the least favored in society.

From the viewpoint of the theory of principles, by Robert Alexy, according to Virgílio Afonso da Silva (34), there is a possibility of conflict between legal rules and of collision between legal principles which make up the fundamental rights. But it is also possible that a collision occurs between rules and principles, which is another way in which a collision between principles may take place (34).

Ronald Dworkin, in his work “Taking Rights Seriously” (36) addresses the issue of balancing law principles and the weighting of them as a need to decide, e.g., hard cases for which there are no decisions made yet but they certainly must be made. Some bases of these themes were previously addressed by the author earlier, in 1967, in an article entitled “The Model of the Rules” (37).

Dworkin establishes, in a way that is also indicated by Alexy, and is vastly accepted in law theory that a conflict between two rules has to be solved at the level of validity, with only one possible answer: one of the rules is valid, and the other rule is not valid.

On the other hand, Alexy indicates that in a collision between two principles, the nature of the collision is based in the “factual supports” of the principles, which includes in it the “protection scope of the principle” and the “governmental intervention” (34).

The “factual support” in Virgílio Afonso da Silva (4), on the other hand, also includes the “constitutional reasoning” in the conception of the constitutional principle to define whether the rights restriction based on rights collision, e.g., is constitutionally permitted or not.

Therefore, the technique to solve rights collision is to weigh the related principles, balance them, and select the one with greater weight, applying the proportionality principle conception to decide which is the most relevant principle to prevail for the governmental action to face the issue (facts) addressed, in order to restrict fundamental rights exercises in a constitutionally respectful pattern. And this weight and importance must be in accordance with the fairness of the decision for that situation (32, 34, 36).

Virgílio Afonso da Silva developed his own concept of the “factual support” based on Alexy's theory to clarify the constitutional permission of the fundamental rights restriction under a necessary verification: Scope of protection of the fundamental right + Governmental intervention + Constitutional Reasoning = Constitutional and Acceptable restriction of a Fundamental Right Exercise, case by case, necessarily considered the proportionality principle (4). If there is no constitutional reasoning for the measure/intervention adopted to restrict fundamental rights, the State's intervention in this case will be unconstitutional.

When two principles collide, e.g., the principle of individual liberty and the principle of public health, it must be assessed which principle should prevail in that specific scenario, considering and deciding in favor of the most fair decision to take (according to Alexy, the maximum protection of the fundamental right), always balancing and describing the weights of the law principles (rights) related to the situation. From the notion of the right to liberty that can be limited, in a Theory of Justice by Rawls, it is considered that liberty can be limited in favor of everyone's own equal liberty (32).

Added to the notion of the emergency of public health, everything indicates that the right to health in the context of the current pandemic must prevail over the right to unrestricted liberty of movement of people, because the health right in this pandemic carries more legal and moral weight than the liberty exercise with some necessary restrictions, considering also that the liberty exercise can never be without any restriction, for the common good of all people.

Thus, the legality and legitimacy of the extreme measures that restrict freedom rights in the strict duty to cope with the COVID-19 pandemic are well-justified if they are based in fairness and on solid facts, better scientific evidence, acceptable rules and constitutional principles, and if they are made by the competent authorities. Those measures can also be accepted if they use the least aggressive and restrictive measures possible to achieve the public health goal, if they do not cause direct or predicable harm to the life, dignity, and health of anybody, if they prioritize the health rights protection of the most vulnerable in the first instance, and if the measure taken is proportional to the risk faced and to the better protection of indivisible and interconnected fundamental rights.

In the Brazilian case, there is a regulation of social restrictions based on Federal and Local normative acts that settles legal rules during the pandemic. These rules inform other normative acts, improved per locality, which can be more restrictive but not more flexible than the Federal rules or the Federal State's rules. Hence, the normative prescriptions can be improved and adapted in local rules, on the level of the State, the Municipal, and the Federal District governments.

Accordingly, a potential collision of norms of broad spectrum of freedom rights exercises in the face of the established rules of health protection, in the context of the pandemic, to better protect health rights and healthcare systems demonstrates the possibility of the collision of a constitutional principle (rights to freedom) with a rule of protection of the right to health (restriction measures).

In this case, as described for similar abstract situations by Virgílio Afonso da Silva disserting on Dworkin's and Alexy's concepts (34), a principle (of liberty) is restricted by a rule (pandemic extreme measures set by law) so that other principles can prevail—in this case the principle of public health (which is the foundation of the right to health).

The Supreme Federal Court in Brazil has several precedents establishing that whenever health and life collide with constitutional principles less important than life (life with dignity is the most important democratic principle) (5), the rights to life and to health must prevail.

The US Supreme court on May 29, 2020 rejected a church challenge to California's COVID-19 restrictions by a 5–4 vote. Chief Justice John Roberts joined the liberals and said in his opinion that he would not join conservative judges escalating efforts to override public health measures in the name of religious freedom. It was set that “the Supreme Court will not facilitate the spread of a deadly virus in the name of the first amendment” (38).

In the context of the pandemic, the acceptable measures to restrict individual and social rights are strictly intended to contain the spread of the SARS-CoV-2 virus always with the aim of preserving public health and people's lives.

In an ethical approach there is the necessity to consider the argument of utilitarianism decisions to achieve major happiness as a finality (principle of happiness), at least to most people. The point defended by John Stuart Mill's utilitarianism (39) can be a point of departure, but not the foundation of the ethical decision. Other important legal and ethical implications must be considered for decision-making during a pandemic.

No life can be forgotten, that is the point. Decisions in such situations that involve freedom rights exercise, health rights access, and protection of healthcare systems during pandemics, must be decisions that do not directly affect anyone with imminent risk of death or health injury, and it must also predict the consequences of extreme decisions, in its foundations for not to put in real and imminent risk the human dignity of any person. For example, an extreme pandemic measure that put someone in imminent risk of death to protect the health right of most people is not a feasible and fair ethical or legal decision.

That is why the “rule utilitarianism” is, till today, a good way to make ethical decisions guided by law, as it establishes that “an act is morally right if and only if it is (or is likely to be) in accordance with an acceptable rule (or set of rules), where the acceptability of a rule is determined on utilitarian grounds” (40).

The present study developed Table 4 with resumed information of the recommendations found within this research for a guideline on taking pandemic measures fairly to the fundamental human rights and based on acceptable ethical rules.

**Table 4:** Guidelines of conditions to orient authorities to take pandemic measures respecting acceptable ethical issues and fundamental human rights.

**Extreme measures and its flexibilizations orders in pandemics must be, concomitantly:**	**BASED IN:** Solid facts **OR** Better scientific evidence **AND** Better protection of all people **OR** Minor public health risks **AND** Minor individual health risks **OR** Prioritization of individual or collective health rights protection	**NOT CAUSE INJURY DIRECTLY TO**: Life **AND** Health **AND** Dignity **OF ANYBODY;** **OR IS LIKELY TO** **(PREDICTABLE TO):** Cause no harm to anybody **AND** Better and in first place favor the most vulnerable	**FAIR IF VALID TO:** All persons in the same conditions **OR** One person or group in specific condition	**TAKEN WITH:** The least aggressive effort to achieve the aimed public health benefits.	**RESPECTFUL TO:** Proportionality between the public health measure taken and the pandemic related risk faced.
Home quarantine					
Off-site quarantine^**^					
Social distancing					
Social isolation^**^					
Compulsory testing^**^					
Compulsory treatment^**^					
Compulsory vaccination^**^					
Person apprehension^*^					
Schools closure					
Schools reopening					
Travel restrictions					
Closure of clinics					
Closure of stores					
Closure of services					
Jail prison order^*^					
Home prison order^*^					
De-hospitalization					
Confinement^*^ detention					
Cities closure					
Contact tracing					
Temperature Checks					
Streets closures					
Roads closure					
Lockdown measures					
Private and health goods/materials apprehensions					
Compulsory use of masks					
Commerce, stores, services reopening					

Table Interpretation: (A)

* *These measures must be decreed by a criminal judge, in the Brazilian law system, after a requirement of a competent public authority, and must be related to a crime investigation on course or related to a conviction settled by a criminal judge (21).* (B)

** These measures can be taken by public administrative authorities due to the COVID-19 exceptional Federal and States legislations in Brazil, but, for a systematic view of fundamental rights, in case of enforcement measures with the use of public force against individuals physical liberty exercise, it is strongly recommended, for involving people's direct subtraction of liberty, to count with an ongoing criminal investigation and legal order emanated by a criminal judge after the occurrence of a crime related to the pandemic theoretically practiced by the individual (except in the cases such as of vaccination and of treatments with no iminent risks to life, that cannot be forced by physical strength). Isolation measures recommended are obligatory but if physical use of force to make it effective is not needed, the judicial order is also not necessary. But further criminal effects can arise if the individual deliberately do not accomplish with the measure determined by the public administrative authority (isolation after testing positive for COVID-19). These recommendations are founded on the due process of law principles and it's criminal law procedure rights and guarantees (21). (C) The measures of compulsory collecting samples and vaccination do not need a medical doctor prescription to be obligatory in Brazil. On the other hand, the measures of medical examinations, laboratorial tests and treatments, and social isolation must be abiding to medical prescription to be obligatory to the individual in Brazil due to the pandemic legislation in effect (30). (D) The other measures in the Brazilian scenario can be made by public administrative action, due to Executive and Legislative authorities' norms, under their competency to legislate and administrate the health subject of law, also with the use of police force. (E) In case that the competent authorities do not act in their public duty or act against it, the Judiciary power can be provoked to act and deliberate in all these measures, regarding to its legal competencies, by court decisions, also founded in the principle of prohibition of the non-liquet (and the principle of no judgement avoidance) according to which the Judiciary Power in Brazil has the duty to decide in all questions that are demanded in courts (21). (F) If the pandemic exceptional/extreme measure and its flexibilization measure has all five affirmatives to the conditions above, the related measure is fair and accomplish with the fundamental human rights of all, aiming to protect and to promote rights to health in a pandemic scenario. In this case, according to the “Rule utilitarianism,” the measure taken in the case fact is also accordant with an acceptable and ethical rule and that is a moral right act also based on utilitarian grounds.

*Source: Data prepared, interpreted from the research findings, and formatted by the authors, from the data related on the references section of this research*.

## Conclusions

It is legal and legitimate for governments to adopt extreme measures by balancing and weighting constitutional principles to adopt restrictions on the fundamental rights exercise that collide with the fundamental rights to health in situations of a pandemic, on the level of the Constitution. It is extremely necessary to protect rights to life, dignity, and the health of all. The prioritization of them in the face of freedom rights divorced from the solidarity, self-protection, care, and respect of autonomy, values that are important to societies, must be considered in health decisions.

To face the collision of rights (on the level of constitutional principles) it is necessary that facts have not yet being legislated specifically by a rule, or the specific rules are imprecise or incomplete, or if the rules (indirectly the principles that forged the rules) collide with other fundamental principles based on the Constitution. For that reason, these situations need the constitutional principles that address fundamental human rights to complement or define the legal significance of the governmental intervention to be applied. Pandemic occurrences must require this special legislation (rules) and precedent rulings of courts to determine the legal conformation of rights restriction measures for decision-making.

The conflict of general legal rules and special rules for a pandemic, at the other side, must be decided at the validity level, in which a pandemic rule must prevail when applied to the current situation, by the application of the prevalence of a special rule instead of a general rule, according to the *Latin sentence lex specialis derogat generali*.

If one intends to achieve a state of social justice during a pandemic, at the same time the government must first protect the most vulnerable. Then it is necessary to protect all people's rights to health and liberty with solidarity, or health equity will not be possible, mainly in impoverished communities, in prisons, and in peripheral regions of the world.

Most of the articles selected in the present study legitimize social restraint measures to face the increase in the number of infected cases in the pandemic by factual evidence of contribution to decreasing the contagion and spread of the virus.

The same direction is noted in Table 3 data, in which seven court decisions face extreme measures related to the pandemic, and in its majority (5–2) they give precedence to the protection of the rights to health related in the case (only the cases 2. and 7., the “unblock of roads” and the “Citizen 3” cases, were not decided invoking the need of the extreme measure adopted to protect the right to health, as described in Table 3).

Nevertheless, there are some selected articles in this study that claim the alleged ineffectiveness or limited effectiveness of containing the transmissibility of the virus by restricting the movement of people, and there are also respectful researches advocating the contradiction of the travel restrictions to the rules of international law. These viewpoints are worth considering despite their limited influence due to the adoption of extreme measures since the SARS pandemic of 2002-2004, but they make an important point by indicating that it is necessary to consider the balance between the health protection of everyone and the preservation of the rights to freedom of all and all related fundamental rights that can be restricted in their exercise by pandemic extreme measures adopted by governments based on scientific evidence.

Restrictive measures must abide by the legal and constitutional systems, the social conditions and must be in harmony with the notion of relativity of fundamental rights. There exists the feasibility that health rights rules must take precedence over general freedom rights in the scope of a pandemic. The present research created guidelines for authorities to take during a pandemic when adopting extreme measures that affect fundamental rights exercises in a way of respecting the fundamental human rights of all and for the consideration of acceptable ethical decisions in this same direction. The guide is summarized in Table 4.

## Author Contributions

JS concepted the theme and idea of the research, indicated a law science author for consideration, and redacted its first manuscript. PS made the research of scientific articles used within this article, selected and interpreted them, selected the law science authors to be cited, concepted the structure of the research, and comparison of data. Also designed the tables, the article type, format, sections, headings and their nomenclatures, the abstract, the keywords, and the others sections of the article. Wrote the final version of the manuscript submitted, prepared the template for the journal submission, and designed its english version that was revised by experienced translators. FA coordinated the research and the writing process of the manuscript. IB, PM, and ET-C analyzed the data collected and suggested modifications and made revisions. LA conducted the process of creation, coordinated the authors, made suggestions in the conception and writing of the manuscript, and contributed within the data analysis. All authors contributed to the article and approved the submitted version.

## Conflict of Interest

The authors declare that the research was conducted in the absence of any commercial or financial relationships that could be construed as a potential conflict of interest.

## Footnotes

^1^Foreword GJ. Rights as trumps? (2018) 132 Harvard Law Review. 28, 96–117.
